# Investigation of the growth performance, blood status, gut microbiome and metabolites of rabbit fed with low-nicotine tobacco

**DOI:** 10.3389/fmicb.2022.1026680

**Published:** 2022-10-13

**Authors:** Changliang Jing, Jiahao Wang, Yi Xie, Jianhui Zhang, Yixuan Guo, Tian Tian, Jing Tang, Fuzhu Ju, Chunkai Wang, Yanhua Liu, Zhongfeng Zhang, Xingyou Yang, Hongbo Zhang

**Affiliations:** ^1^Key Laboratory of Synthetic Biology of Ministry of Agriculture and Rural Affairs, Tobacco Research Institute of Chinese Academy of Agricultural Sciences, Qingdao, China; ^2^Sichuan Tobacco Science Research Institute, Chengdu, China; ^3^College of Animal Science and Technology, Qingdao Agricultural University, Qingdao, China; ^4^Institute of Animal Science of Chinese Academy of Agricultural Sciences, Beijing, China

**Keywords:** low-nicotine tobacco leaf, rabbit, microbial community, blood parameters, metabolites

## Abstract

Tobacco contains a large amount of bioactive ingredients which can be used as source of feed. The objective of this study was to evaluate the effects of dietary addition of low-nicotine tobacco (LNT) on the growth performance, blood status, cecum microbiota and metabolite composition of meat rabbits. A total of 80 Kangda meat rabbits of similar weight were assigned randomly as four groups, and three of them were supplemented with 5%, 10%, and 20% LNT, respectively, with the other one fed with basal diet as control group. Each experiment group with 20 rabbits was raised in a single cage. The experiments lasted for 40 days with a predictive period of 7 days. The results revealed that LNT supplementation had no significant effect on the growth performance, but increased the half carcass weight compared with control group. Dietary supplemention of LNT decreased the triglycerides and cholesterol content in rabbit serum, and significantly increased the plasma concentration of lymphocytes (LYM), monocytes, eosinophils, hemoglobin HGB and red blood cells. In addition, LNT supplementation significantly changed the microbial diversity and richness, and metagenomic analysis showed that LNT supplementation significantly increased *Eubacterium_siraeum_group*, *Alistipes*, *Monoglobus* and *Marvinbryantia* at genus level. Moreover, LC–MS data analysis identified a total of 308 metabolites that markedly differed after LNT addition, with 190 significantly upregulated metabolites and 118 significantly downregulated metabolites. Furthermore, the correlation analysis showed that there was a significant correlation between the microbial difference and the rabbit growth performance. Overall, these findings provide theoretical basis and data support for the application of LNT in rabbits.

## Introduction

Tobacco (*Nicotiana tabacum* L.) is a commercial crop grown worldwide. In addition to being used for cigarettes, tobacco is also one of the plant resources containing a sufficient quantity of vitamins, minerals and proteins meeting with the nutrition demands of animals (Tabe and Higgins, 1998). Moreover, tobacco is a rich source of different bioactive compounds with multiple biological activities, especially abundant in phenolic compounds, such as chlorogenic acid, kaempferol, caffeic acids, rutin, isoquercetin, and luteolin (Ru et al., 2012; Sifola et al., 2021). It can also synthesize a variety of beneficial components or drug precursors, such as cembrane diterpenes with antioxidant functions (Xu et al., 2022). The above features indicate that tobacco has great potential for further development and utilization. However, nicotine in tobacco is the key component that limite the development and utilization of tobacco for multiple purposes. Even though nicotine exhibits an inhibitory effect on Alzheimer’s disease and Parkinson’s disease by activating the acetylcholine receptors and protecting nerves, long-term exposure to high doses of nicotine can cause certain damage to the immune system and depress the central nervous system (Riljak and Langmeier, 2005; Barreto et al., 2015). Our previous study reported the development of LNT with animal feeding potentials and made it feasible to expand the multi-purpose utilization of tobacco (Wang et al., 2022).

With the rapid development of animal industry, the prices of raw materials for conventional feed are rising, and the cost of breeding continues to increase. Mining and broadening unconventional feed resources have become key measures to promote the sustainable and healthy development of animal production (Karlsson and Röös, 2019). The high-level of protein, sugar, polysaccharides and other bioactive ingredients in tobacco make it with potentials for animal production (Banožić et al., 2019). And, the favorable impacts of tobacco have been investigated as protein resource for piglets (Rossi et al., 2013, 2014). However, there are few reports on the use of LNT as feed materials for meat rabbits. In our previous studies, it was found that fresh tobacco leaves with nicotine content of <0.3% can be used for feeding (Wang et al., 2022). The appropriate addition amount and safety evaluation of LNT in meat rabbit diet are yet to be studied.

Based on the previous studies, this work utilized LNT as a feeding resource for meat rabbits and investigated the effects on rabbit growth and other performance. By adding different proportions of LNT to the daily diet of meat rabbits, the growth performance, slaughter performance, meat quality, cecum flora and metabolites of rabbits were inspected. The results of this study would enlighten the knowledge about the effects of LNT tobacco on animal growth and contribute to the application of LNT as a novel resource for animal production.

## Materials and methods

### Ethics statement

This study was strictly carried out following the regulations for experimental animals of the China Department of Agriculture and approved by the Animal Care and Use Committee of Qingdao Agricultural University (QAU2021-0455).

### LNT and feed preparation

LNT (CD01, a low-nicotine tobacco derived from the crossing progenies of tobacco K326 and ULA-Hi) was cultivated by Tobacco Research Institute of Chinese Academy of Agricultural Sciences and planted in Jimo experimental station, Qingdao (N36°26′53.1155″, E120°34′38.0317). The temperature ranged between 15°C and 35°C (average 28.5°C) and the higher temperature typically occurred between 1:00 and 3:00 p.m. The relative humidity averaged 51%. Leaves were harvested after maturity and then dried by heating at 60°C. The ingredients and nutrient content (%) of LNT are listed in Supplementary Table S1. The diets of rabbits were formulated according to the NRC. The composition and nutrition of diets are shown in Supplementary Table S2. The control group was fed the basal diet, and the experimental groups were fed the full-price compound diet prepared with 5%, 10%, and 20% LNT, respectively.

The nicotine content in tobacco leaves and feed was measured as following. Samples were dried at 65°C and grounded into powder, and 0.1 g of sample was weighed for each measurement. The sample powder was added to a 50 ml glass centrifuge tube, and 1 ml of 10% sodium hydroxide solution and 5 ml of ethyl acetate containing internal standard (2, 4-bipyridine) were then added. After mixing by vortex, the extract was ultrasonically extracted at 40°C for 15 min, kept at room temperature for overnight extraction, and then centrifuged at 3,000 rpm for 10 min and filtered the supernatant through a 0.22 μm filter. Nicotine measurement was performed using a GC–MS machine equipped with an HP-5MS capillary column (30 m × 0.25 μm × 0.25 μm). A serial of nicotine standard solutions were used to plot the standard curve for GC–MS detection, and the nicotine content in samples was calculated based on the standard curve.

### Animals, housing, and treatment

For the feeding experiment, 80 of 45-day-old meat rabbits (Kangda meat rabbit commodity generation) with similar body weight (750 ± 10.4 g) were obtained from Qingdao Kangda Meat Rabbit Development Professional Cooperative and randomly divided into four groups, with five replicates for each experimental group and four rabbits (half male and half female) for each replicate. Each replicate was raised in a 0.6 m × 0.7 m × 0.5 m clean cage. There were no significant differences in the body weight (*p* > 0.05). The diets of each treatment were processed into pellets and fed two times a day. Before starting the experiment, the rabbit house and cage were cleaned and disinfected, and the rabbit house was naturally ventilated and lighted according to the routine procedures. Feeding and free water at 08:00 and 17:00 every day. The pre-trial period is 7 days and the experimental period was 42 days. The rabbit shed temperature was maintained at 15°C–25°C.

### Sample collection and preparation

One night of fasting (drinking water freely) was set before the end of the experiment. At the end of the experiment, 10 meat rabbits (half male and half female) with similar body weights were selected for slaughter, weighed and recorded before slaughter. Blood samples from the rabbit of each replicate were randomly collected by cardiac puncture into vacuum tubes containing an anticoagulant and centrifuged at 3,000 rpm for 10 min at 4°C. Pure plasma samples were collected and stored in 1.5 ml eppendorf tubes at −20°C. The digesta samples in the cecum were stored in sterile cryopreservation tubes and frozen immediately at –80°C for further analysis.

### Determination of slaughter performance index

At the beginning and the end of the experiment, the body weight of each group on empty stomach was measured at 8:00 a.m., and the initial body weight (IBW), final body weight (FBW) and average daily gain (ADG) were determined. The average daily feed intake (ADFI) was inspected and the feed-to-weight ratio (F/G) was calculated according to ADG and ADFI. The number of rabbits with diarrhea and the number of dead rabbits were recorded during the experiment, and the diarrhea rate and mortality were calculated.

Followings are equations for above calculations: ADG = (FBW-IBW)/(test days in the positive trial period × the number of rabbits in the repeat test); ADFI = total feed intake per repeated positive trial period/(test days in the positive trial period × the number of rabbits in the repeat trial); F/G = ADFI/ADG; diarrhea rate (%) = (the number of rabbits with diarrhea/total number of experimental rabbits) × 100; mortality (%) = (the number of dead rabbits/total number of experimental rabbits) × 100.

The whole evisceration is the weight of the carcass after slaughtering with blood, fur, head, tail, forelegs, hindlimbs and all internal organs removed. The weight of half evisceration and the weight of full evisceration, which were discriminated by the live weight before slaughter, were the slaughter rate of half evisceration and the slaughter rate of full evisceration, respectively.

### Determination of blood physiological and biochemical indicators

Before the end of the experimental period, whole blood and 5 ml of dipotassium ethylenediaminetetraacetate (EDTA-K2) for anticoagulation were collected from the experimental rabbits after overnight fasting. The collected serum was stored at −80°C for further study. The anticoagulant was placed at room temperature, and blood routine indexes were measured after 3 h.

White blood cells (WBC), neutrophils (NEU), lymphocytes (LYM), monocytes (MON), eosinophils (EOS), basophils (BAS), the medium percentage of neutrophils (NEU), the percentage of lymphocytes (LYM), the percentage of monocytes (MON), the percentage of eosinophils (EOS), the percentage of basophils (BAS), the number of red blood cells (RBC), the hemoglobin (HGB), hematocrit (HCT), mean corpuscular volume (MCV), mean corpuscular hemoglobin content (MCH), mean corpuscular hemoglobin concentration (MCHC), red blood cell width coefficient of variation (RDW-CV), red blood cell distribution width standard deviation (RDW-SD), platelet number (PLT), mean platelet volume (MPV), platelet distribution width (PDW), platelet volume (PCT) and other blood routine indicators were determined by German ABX blood cell analyzer.

For biochemical index determination, the rabbit blood was collected and placed in a 10 ml vacuum blood collection tube (containing heparin sodium). After standing at room temperature for 4 h, the serum was prepared by centrifugation at 3,500 rpm for 10 min. Then, an automatic biochemical analyzer was employed to determine the biochemical index. The determined indicators include alanine aminotransferase (ALT), total protein (TP), albumin (ALB), urea (UREA), globulin (GLOB), creatinine (CREA), uric acid (UV), cholesterol (CHOL), alkaline phosphatase (ALP), γ-glutamyltransferase (γ-GT), glucose (GLU), lactate dehydrogenase (LDH), Ca and P content.

### Meat quality determination of muscle physical properties

After the tested rabbits were slaughtered, the longissimus dorsi muscle of rabbit was collected, and the color of rabbit meat [redness (a*), yellowness (b*) and brightness (L*)], drip loss and pH were determined according to previous methods (Liu B. et al., 2022).

### DNA extraction and 16S rRNA gene sequencing

Microbial DNA was extracted from rabbit cecum samples by CTAB/SDS. DNA concentration and purity were determined using a Fisher NanoDrop 3300 UV–visible light spectrophotometer (Thermo Scientific, United States), and DNA quality was checked by electrophoresis in 1% agarose gel. The DNA samples were diluted to 1 μg/μl using sterile water according to the DNA concentration. The microbial 16S rRNA/18S rRNA/ITS genes was amplified by PCR using specific primers (such as 16SV4:515SVR-106SV4-18S, 18SV4/18S, 18SV4/18SV9, ITS1/ITS2, 18SV4:528F-18SV9F-1510R, etc.). The thermal cycling procedure included an initial denaturation at 98°C for 1 min; 27 cycles of denaturation at 98°C for 10s, annealing at 50°C for 30s, and extending for 30s at 72°C; and a final extending step at 72°C for 10 min. Three replicates were performed for each PCR amplification, and all reactions were performed using 15 μl Phusion^®^ High-Fidelity PCR Master Mix (New England Biolabs), 0.2 μM forward and reverse primers, and ~10 ng of template DNA. The PCR reaction product was mixed with the same volume of loading buffer and separated by electrophoresis in 2% agarose gel. Gel extraction of the PCR products was performed using the Qiagen Gel Extraction Kit (Qiagen, Germany). Sequencing libraries were generated using TruSeq^®^ DNA PCR-Free Sample Preparation Kit (Illumina, United States) with added index code, and evaluated by Qubit^@^2.0 Fluorometer (Thermo Fisher Scientific, Germany). Finally, the qualified library was sequenced with 250 bp paired-end reads on Illumina NovaSeq platform (Illumina, United States).

### Untargeted metabolomics study based on liquid chromatography tandem mass spectrometry

Rabbit cecal metabolites were analyzed by Vanquish UHPLC system (Thermo Fisher Scientific, Germany) coupled with an Orbitrap Q Exactive™ HF mass spectrometer (Thermo Fisher Scientific, Germany) in Novogene Co., Ltd. (Beijing, China). The cecal sample (1 ml) was freeze-dried and resuspended with precooled 80% methanol by vortexing. Then, the samples were incubated on ice for 5 min and centrifuged at 15,000*g* for 10 min at 4°C. A certain volume of the supernatant was diluted in LC–MS grade water to a final methanol concentration of 53% and then transferred into a new Eppendorf tube. The samples were subsequently centrifuged at 15,000*g* for 15 min at 4°C to collect the supernatant for further determination.

Sample for detection was injected into a Hypesil Gold column (100 × 2.1 mm, 1.9 μm) employing a 17 min linear gradient at a flow rate of 0.2 ml/min. The eluent for positive polarity mode was composed of 0.1% FA (eluent A) and methanol (eluent B), and that for negative polarity mode was composed of 5 mM ammonium acetate (pH 9.0, eluent A) and methanol (eluent B).The solvent gradient was 2% B for 1.5 min, 2%–100% B for 3 min, 100% B for 10 min, 100%–2% B for 10.1 min, and then 2% B for 12 min. Q Exactive™ high-frequency mass spectrometer was operated with spray voltage 3.5 kV in positive and negative polarity mode, with the capillary temperature of 320°C, sheath gas flow rate of 35 psi, auxiliary gas flow of 10 L/min, S-lens RF level of 60, and auxiliary gas heater temperature of 350°C.

The raw data generated by UHPLC–MS/MS were processed using Compound Discoverer 3.1 (Thermo Fisher Scientific, Germany) for picking and quantifying the peaks for each metabolite. The data after normalization to the total spectral intensity were used to predict the molecular formulas based on additive ions, molecular ion peaks, and fragment ions, and the accurate and relative quantitative results were obtained by matching the peaks with mzCloud,^1^ mzVault and MassList databases. Statistical analyzes were performed using the statistical software R 3.4.3, Python 2.7.6, and CentOS 6.6. The abnormally distributed data were transformed using area normalization method.

### Statistical analysis

Statistical analyzes were performed using SPSS 20.0 software. Differences between the values were assessed using Duncan’s test, and that of *p* < 0.05 was considered statistically significant. The correlations between the cecum microbial composition (relative abundance of genus higher than 0.1%) and the metabolites that were significantly affected by LNT addition were assessed by a Spearman’s correlation analysis using GraphPad Prism version 8.00 (GraphPad Software, United States).

## Results

### Growth performance and diarrhea incidence

As shown in Table 1, addition of different LNT had no significant effect on the final body weight, average feed to weight ratio, diarrhea rate, mortality rate, whole carcass weight, whole carcass ratio, and half carcass rate of rabbits (*p* > 0.05) compared with control. Interestingly, the addition of LNT significantly increased the half carcass weight (*p* < 0.05) of rabbits, and this effect was more significant in the 20% LNT addition group (Table 1). These results suggested that LNT has potential to be developed as a feed resource without harm to rabbits.

**Table 1:** Effects of LNT on growth performance and diarrhea incidence of meat rabbits.

Index	LNT addition percentage/%				*p*-value
	0%	5%	10%	20%	
IBW/g	0.76	0.76	0.74	0.76	0.785
FBW/g	2.62	2.56	2.67	2.70	0.183
ADG/(g/d)	41.48	40.37	42.96	43.18	0.156
F/G	2.18	2.23	2.10	2.09	0.150
Diarrhea rate/%	1	0	1	0	–
Mortality rate/%	1	0	1	0	–
Whole carcass weight/g	1.69	1.76	1.75	1.75	0.057
Whole carcass ratio/%	64.82	68.68	65.45	64.76	0.062
Half carcass weight/g	1.33^c^	1.35^b^	1.39^b^	1.41^a^	0.016
Half carcass ratio/%	50.73	52.72	52.09	52.03	0.407

IBW, initial body weight; FBW, final body weight; ADG, average daily gain; F/G, feed/gain. Superscript letters within each row indicate significant difference between the group (*p* < 0.05).

### Blood biochemical parameters

As shown in Table 2, the serum triglycerides (TG) and cholesterol (CHO) content of meat rabbits were significantly lower compared with control, and the serum TG decreased along the increase of LNT supplementation (*p* < 0.05). Adding LNT to the rabbit diet could significantly increase the number of lymphocytes, the percentage of lymphocytes, the percentage of monocytes, the percentage of eosinophils, the content of hemoglobin HGB, and the percentage of red blood cells in the blood of rabbits (*p* < 0.05).

**Table 2:** Effects of LNT on blood biochemical parameters of rabbits.

Index	LNT addition percentage/%				*p*-value
	0	5	10	20	
TG	0.98^A^	0.85^A^	0.68^A^	0.47^B^	0.002
CHO	2.87^a^	1.25^c^	2.18^b^	1.84^b^	0.047
LYM%	39.70^b^	45.33^a^	46.50^a^	41.47^ab^	0.029
MON%	10.67^ab^	8.93^ab^	7.50^b^	11.70^a^	0.033
EOS%	1.33^b^	1.73^ab^	1.77^a^	1.83^a^	0.035
RBC (×10^12^/L)	5.44^c^	6.40^b^	7.14^a^	6.70^ab^	0.021
HGB (g/L)	121.67^c^	129.33^bc^	146.67^a^	141.67^ab^	0.036
HCT%	36.47^b^	39.57^ab^	44.00^a^	43.27^ab^	0.040
RDW-CV%	12.80^ab^	12.87^b^	12.87^ab^	14.00^a^	0.042
RDW-SD (fL)	33.17^b^	31.33^b^	31.50^b^	35.53^a^	0.012

TG, triglycerides; CHO, Cholesterol; NEU, neutrophil number; LYM, lymphocyte number; MON, monocytes number; EOS, Eosinophil number; RBC, red blood cells; HGB, hemoglobin; HCT, hematocrit; RDW-SD, red blood cell distribution width - standard deviation. Different superscript letters within each row indicate significant difference between the group (uppercase letters for *p* < 0.01, lowercase letters for *p* < 0.05).

### Meat quality of meat rabbits

As shown in Table 3, adding LNT to rabbit diet significantly increased the brightness of rabbit meat (*p* < 0.05), while having no significant effect on the redness, yellowness, pH and drip loss of rabbit meat (*p* > 0.05).

**Table 3:** Effect of LNT on meat quality of meat rabbits.

Index	LNT Addition percentage/%				*p*-value
	0	5	15	20	
Redness	2.62	2.653	2.646	2.713	0.228
Yellowness	4.09	4.113	4.116	4.2	0.455
Brightness	48.873^a^	51.8^b^	52.566^b^	54.176^c^	0.017
pH	6.63	6.593	6.66	6.72	0.07
Drip loss	2.376	2.42	2.413	2.35	0.285

Superscript letters within each row indicate significant difference between the group (*p* < 0.05).

### Diversity, richness, and composition of bacterial communities in cecal content

To examine the cecum microbial community structure, 16S rRNA sequencing was performed to analyze the cecum contents of both groups (control and 20% LNT addition). The dilution curve of deep sequencing of regions V3–V4 flattened as the detection sequence increased and the sequencing data reached saturation (Supplementary Figure S1).

In terms of alpha diversity, no differences in observed species, Chao 1, ACE index, Shannon and Simpon were noted in the two groups (Supplementary Figure S2). By beta-diversity analysis, the principal component analysis (PCA) results showed that they were significantly separated in the first axis (PC_1_ = 13.18%) and the second principal component (PC_2_ = 13.14%). Similarly, the results of NMDS analysis was similar to the PCA analysis, and two group were separated in the first dimension. Further communities were differentially analyzed by the adonis and anosim function in the vegan package, the results of adonis (*p* = 0.003, *R*^2^ = 0.23) and anosim (*p* = 0.008, *R*^2^ = 0.45) indicated significant differences in the cecum microbial community composition between the two groups.

**Figure 1:**
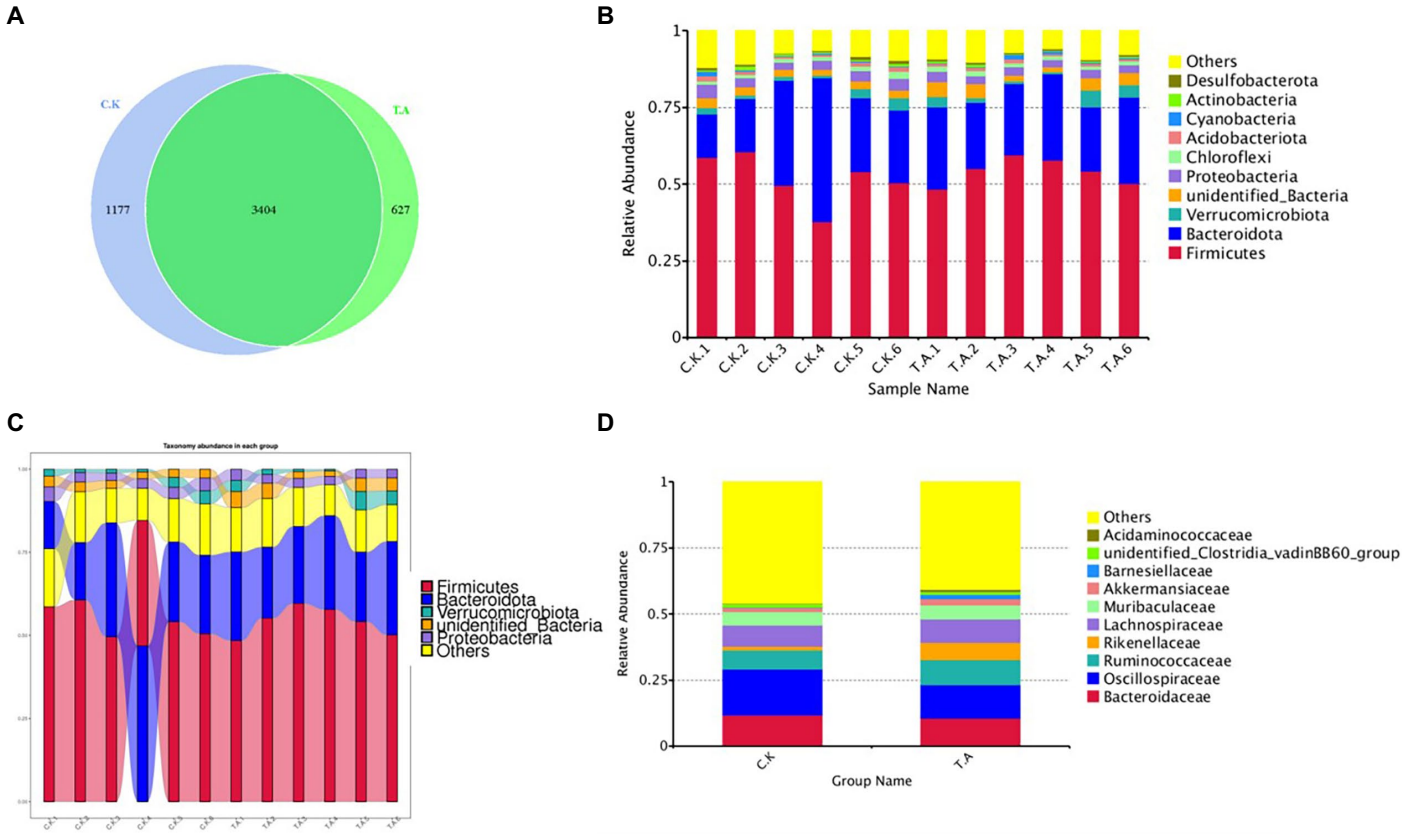
Analysis of the microbial diversity composition of rabbits based on 16SRNA sequencing technology. **(A)** Venn diagram of cecal microbial diversity in two groups. **(B)** Bar graph of the relative abundance of the top10 species. **(C)** Analysis of microbial abundance of meat rabbits on phylum classification. **(D)** Sankey of the microbial abundance of control and LNT (TA).

Community structure composition analysis of the two groups was shown in Figure 1A. A total of 5,208 OTU were found in two group, and after excluding 3,404 common OTU in both treatments, 627 OTU were found in the LNT group and 1,177 in control. There was no statistical difference in the number of OTU between the two groups. At phylum level, Firmicutes (61.2 ± 1.12%), Bacteroidota (31 ± 1.25%) and Proteobacteria (5 ± 0.25%) constitute the core flora (relative abundance >90%; Figures 1B,C). Further comparison showed that Bacteroidota was about 4.2% higher than in the LNT group. However, the Firmicutes in the LNT group were 1.2% higher than that in the control group (Figure 1D).

**Figure 2:**
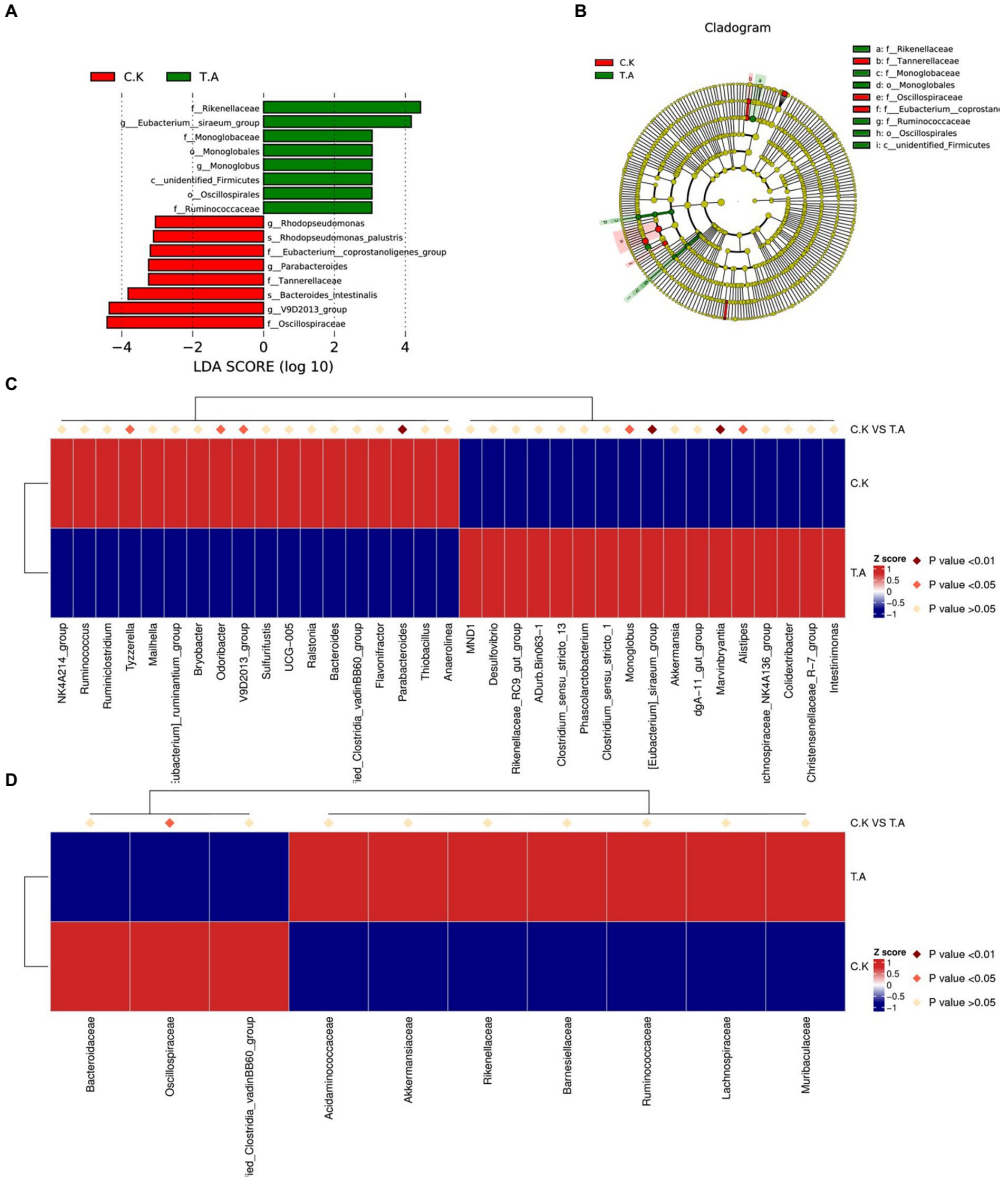
Analysis of cecum microbial community composition differences in rabbits. **(A)** Bar chart of the distribution of LDA values of species with significant differences between control and LNT (TA) (*p* < 0.05, LDA > 3). **(B)** Phylogenetic tree plot of significantly different species between control and LNT (TA). **(C)** Heatmap of differential species between control and LNT (TA) at genus level. **(D)** Heatmap of differential species between control and LNT (TA) at family level.

Analysis of the top 30 abundant families revealed that *Rikenellaceae, Monoglobaceae, Barnesiellaceae, Monoglobaceae* and *Ruminococcaceae* were significantly increased by LNT addition (*p* < 0.05), whereas the abundance of *Ruminococcaceae, Tannerellaceae, Marinifilaceae, Eubacterium_Coprostanoligenes_group* and *Oscillospiraceae* were significantly decreased (*p* < 0.05). Results of the LEfSe analysis are shown in Figure 2. Microbial differences in two groups at genus levels were further analyzed by *t*-test. Compared with control, two genera *Eubacterium_siraeum_group* and *Monoglobus* were significantly enriched in LNT (*p* < 0.05, LDA > 3), while three genus *Rhodopseudomonas, Parabacteroides* and V9D2013_group were significantly decreased after LNT addition (*p* < 0.05, LDA > 3).

### Metabolic spectrum analysis of cecum of meat rabbits

**Figure 3:**
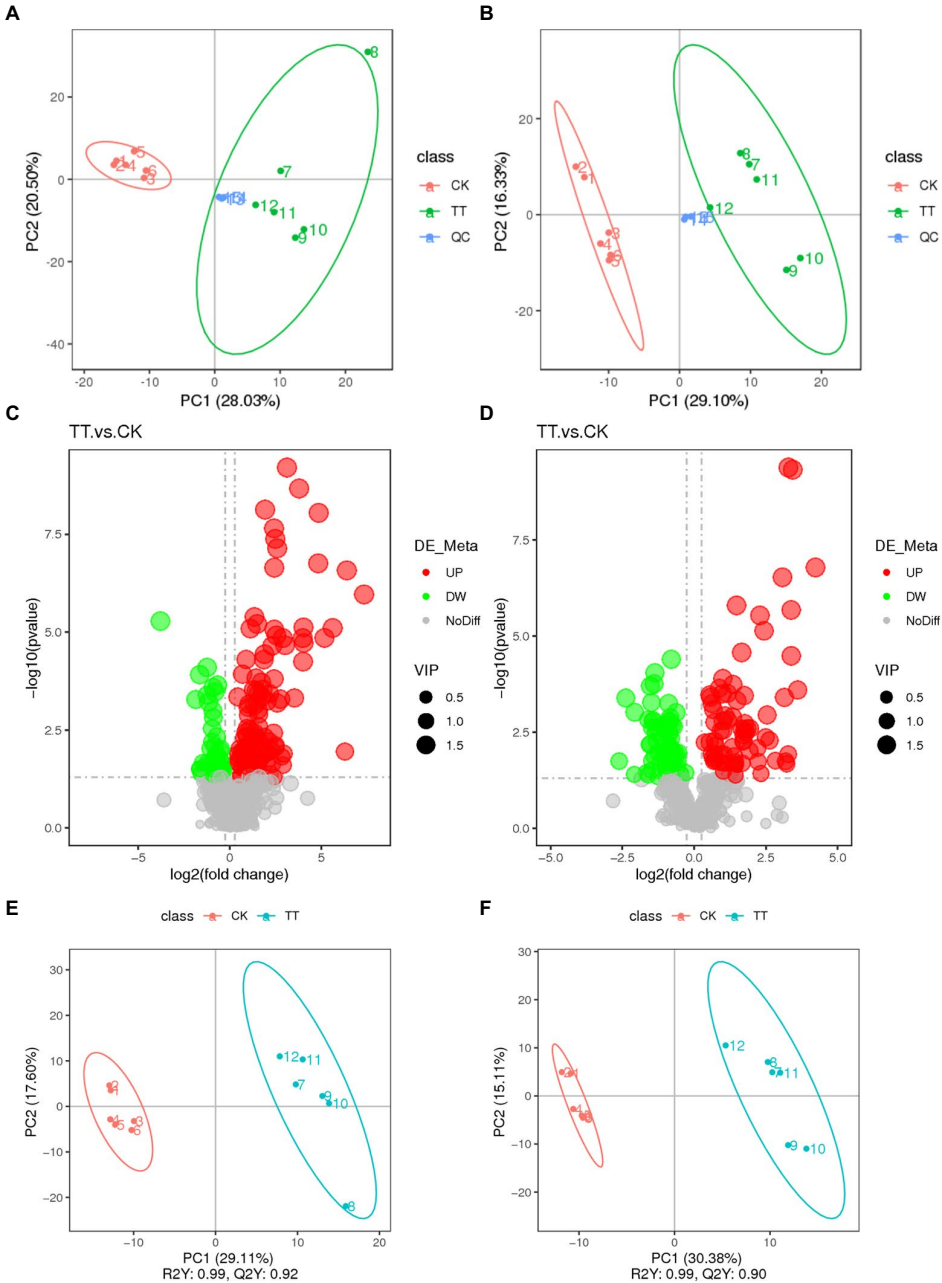
Multivariate statistical analysis of untargeted metabolomics data obtained using the LC–MS/MS approach. PCA score plot of cecum metabolomics data for control and LNT addition (TT) obtained by **(A)** LC-MS (ESI−) and **(B)** LC-MS (ESI+) (*n* = 6). **(C)** PLS-DA score plot of cecum metabolomics data obtained by LC-MS (ESI−); R^2^Y = 0.99; Q^2^Y = 0.90. **(D)** PLS-DA score plot of cecum metabolomics data obtained by LC-MS (ESI+); R^2^Y = 0.99; Q^2^Y = 0.92. **(E)** Score plot of LC-MS (ESI−) data signals detected. **(F)** Score plot of LC-MS (ESI−) data signals detected. Red circles in volcano plots are model-separated metabolites following the conditions of VIP >1.

To further study the effect of LNT on rabbit health, cecum digesta metabolic profiles of two group (control and 20% LNT addition) was acquired by LC–MS. As shown in Figure 3, the two groups were significantly separated from the first principal component with PC_1_ = 28.03%, PC_2_ = 20.50% in positive ion mode and PC_1_ = 29.10%, PC_2_ = 16.33% in negative ion mode. In summary, a total of 949 metabolites were detected, of which 308 were differential metabolites. Furthermore, a total of 74 metabolites in positive mode and 116 metabolites in negative mode were upregulated, and 68 metabolites in positive mode and 50 metabolites in negative mode were downregulated (Figure 3). Quality of the model was later examined by PLS-DA analysis. The R^2^Y and Q^2^Y of the two groups were 0.99 and 0.92 in positive ion mode and 0.99 and 0.90 in negative ion mode, respectively.

In negative ion mode (Supplementary Figure S3), compared with control, 74 metabolites were significantly decreased after LNT addition. The top 10 metabolites (fold change) were 19 (R) -Hydroxy prostaglandin F1α (18.77), Saccharin (12.21), Thromboxane B1 (10.82), Argininosuccinic acid (10.44), Tetradecanedioic acid (10.42), 13,14-dihydro Prostaglandin F1α (9.75), N1-[4-(aminosulfonyl)phenyl]-2,2-dimethylpropanamide (9.69), Adenosine 3′,5′-cyclic monophosphate (9.35), D(+)-Phenyllactic acid (9.11), and 13,14-dihydro-15-keto-tetranor Prostaglandin D2 (8.49). And, 68 metabolites were significantly elevated, the top 10 metabolites (fold change) were 7-Hydroxy-4-chromone (0.82), 3-(3-Methoxyphenyl)propionic acid (0.72), N1-(3-pyridyl)-2,3,4,5,6-pentamethylbenzene-1-sulfonamide (0.69), 13,14-Dihydro-15-keto-tetranor prostaglandin F1α (0.68), Ursolic acid (0.67), 7-Ketodeoxycholic acid (0.65), Cholic acid (0.64), Lysopc 14:0 (0.61), N-Acetyl-Asp-Glu (0.61), and 1-[(1R,2S,3R,5R)-5-Cyclohexyl-2,3-dihydroxycyclopentyl]-3-ethylurea (0.61).

In the positive-ion mode (Supplementary Figure S4), 50 metabolites were significantly elevated and the top 10 metabolites (fold change) were Monolaurin (162.61), All-Trans-13, 14-Dihydroretinol (84.55871319), Prostaglandin J2 (78.84), N-(4-butyl-2-methylphenyl)-N′-[4-(4-methylpiperazino)phenyl]urea (49.57), Docosahexaenoic acid (36.21), 15-OxoEDE (29.15), (+/−)11(12)-EET (28.59), 1-allyl-4,5-diphenyl-2-(2-thienyl)-1H-imidazole (16.51), (+/−)11(12)-DiHET (16.36), and (2E, 4E)-N-[2-(4-hydroxyphenyl)ethyl]dodeca-2,4-dienamide (16.10). There were 116 metabolites significantly decreased, and the top 10 metabolites (fold change) were Taurocholic acid (0.79), p-Mentha-1,3,8-triene (0.75), Hexadecanamide (0.74), Sphinganine (0.72), gamma-Tocopherol (0.73), Oleoyl ethylamide (0.72), Ecgonine (0.71), D-Proline (0.71), Mupirocin (0.70), and (2E,4E)-N-(2-methylpropyl)deca-2, 4-dienamide (0.69).

**Figure 4:**
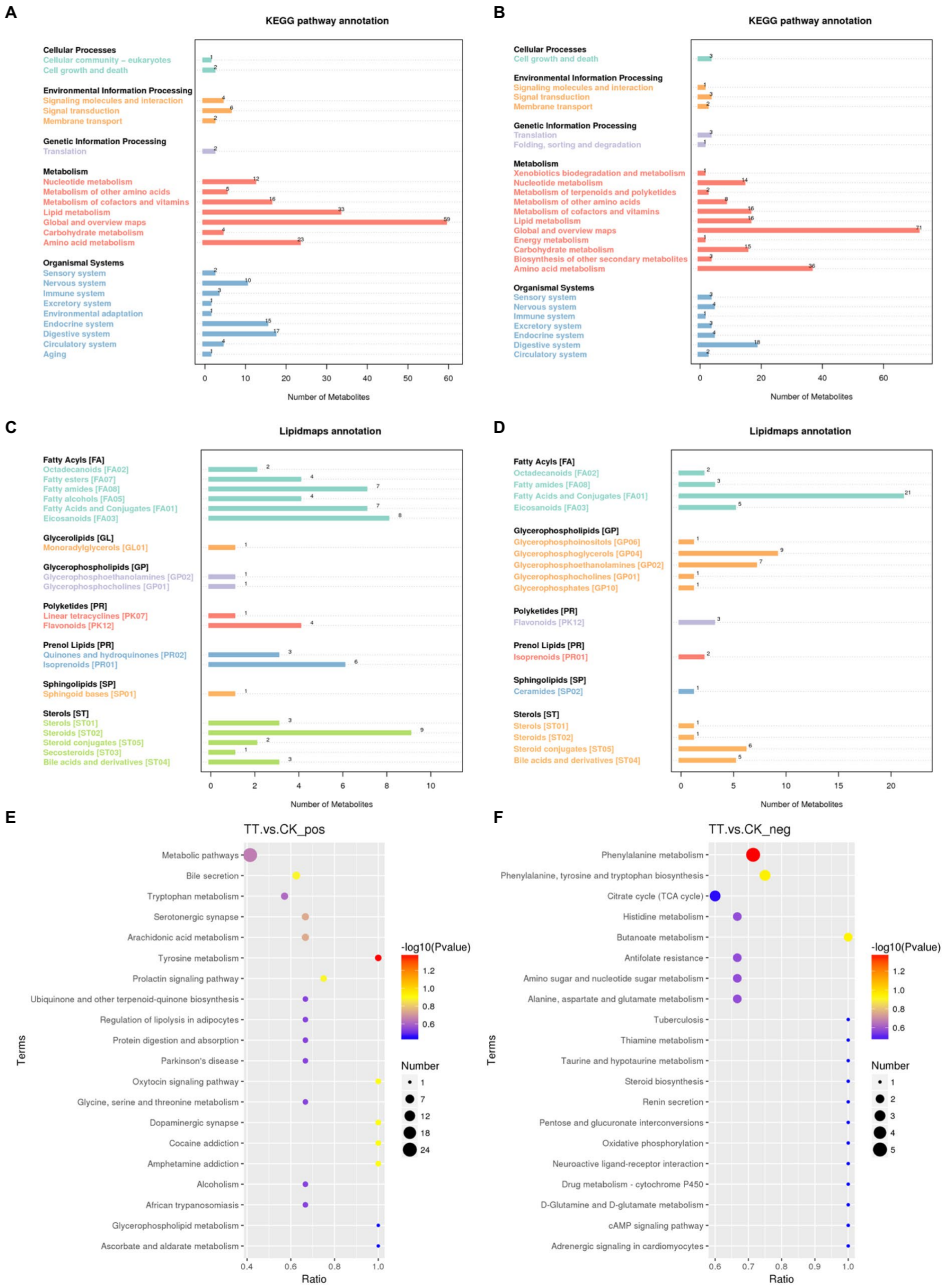
Metabolic pathways enriched in the cecum. **(A,B)** KEGG enrichment analysis. **(C,D)** LIPID MAPS enrichment analysis. **(E)** KEGG enrichment analysis in positive ion mode. **(F)** KEGG enrichment analysis in negative ion mode.

The detected differential metabolites were screened and functionally annotated by KEGG database. There were 33 and 23 metabolites associated with lipid metabolism in control and LNT group, respectively (Figures 4A,B). Further enrichment of lipid-associated metabolic pathways were shown in Figures 4C,D, the top 5 ranked in positive ion mode were Steroids, Eicosanoids, Fatty Acids and Conjugates, Fatty amides, and lsoprenoids. In anion mode, the top 5 pathways were Fatty Acids and Conjugates, Glycerophosphoglycerols, Glycerophosphoethanolamines, Steroid conjugates, and Bile acids and derivatives. Next, according to our Kyoto Encyclopedia of Genes and Genomes (KEGG) pathway analysis, Phenylalanine metabolism (ESI-) and Tyrosine metabolism (ESI+) differed significantly between the two groups (*p* < 0.05) (Figures 4E,F).

### Growth performance-microbiome-metabolome association analysis

**Figure 5:**
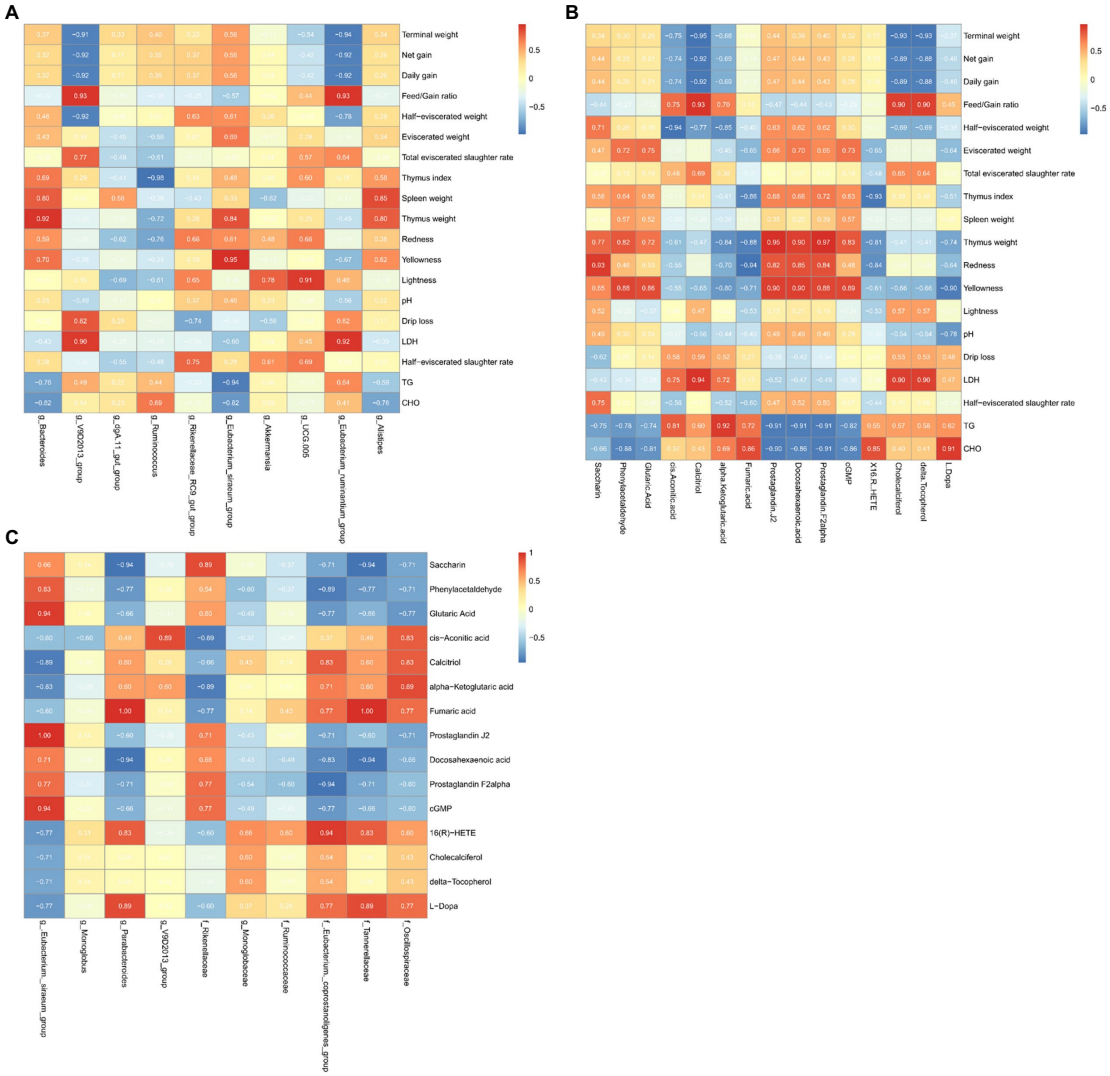
Heat map of microbe-metabolome-growth performance associations in the cecum. **(A)** Microbial enrichment and differential metabolites with >1% enrichment in the cecum. **(B)** Differential metabolites and growth performance. **(C)** Differential microorganisms and differential metabolites.

Spearman correlation analysis of the growth performance, the microorganisms with more than 1% abundance, differential microbes and metabolites was performed, and the correlation coefficient was obtained and expressed as a heatmap (Figure 5).

We found that the microorganisms enriched by >1% were significantly correlated with the meat rabbit growth performance, serum indicators, and meat quality indicators. Among them, *Bacteroides* was positively correlated with Thymus weight (*ρ* = 0.05). *V9D2013_group* was positively associated with LDH (*ρ* = 0.90) and Feed/Gain ratio (*ρ* = 0.93). There were positive associations between *Eubacterium siraeum* with Yellowness (*ρ* = 0.95), *UCG005* with Lightness (*ρ* = 0.91), and *Eubacterium_ruminantium_group* with LDH (*ρ* = 0.92) and Feed/Gain ratio (*ρ* = 0.93). However, *V9D2013* group and *Eubacterium_ruminantium* were all negatively correlated with Terminal weight (*ρ* = −0.91, *ρ* = −0.94), Net gain (*ρ* = −0.92, *ρ* = −0.92), and Daily gain (*ρ* = −0.92, *ρ* = −0.92). *Ruminococcus* was negatively correlated with the Thymus index (*ρ* = 0.98).

In the correlation analysis of metabolites and meat rabbit growth performance, serum indicators, and meat quality indicators. Calcitriol was positively associated with Feed/Gain ratio (*ρ* = 0.93) and LDH (*ρ* = 0.94) and negatively associated with Terminal weight (*ρ* = 0.95), Net gain (*ρ* = 0.92) and Daily gain (*ρ* = 0.92). The lipid-related metabolite: Prostaglandin J2, Docosahexaenoic acid, Prostaglandin were positively associated with Thymus weight (*ρ* = 0.95, *ρ* = 0.90, *ρ* = 0.97) and negatively with TG (*ρ* = −0.91, *ρ* = −0.92, *ρ* = −0.91) and CHO (*ρ* = −0.92, *ρ* = −0.86, *ρ* = −0.91). The cis.Aconitic.acid was negatively associated with half eviscerated weight (*ρ* = −0.94).

In the correlation of differential microbes and metabolites, we found that at the genus level, *Eubacterium._siraeum_group* showed a positive correlation with Glutaric Acid (*ρ* = 0.94), Prostaglandin J2 (*ρ* = 1.00), and cGMP (*ρ* = 0.94). *Parabacteroides* was positively associated with Fumaric acid (*ρ* = 1.00) and negatively associated with Docosahexaenoic acid (*ρ* = −0.94). At the family level, *Eubacterium._coprostanoligenes _group* was positively associated with 16(R)-HETE (*ρ* = −0.94) and negatively associated with Prostaglandin F2alpha (*ρ* = −0.94). Tannerellaceae was positively associated with Fumaric acid (*ρ* = 1.00) and negatively associated with Saccharin (*ρ* = −0.94) and Docosahexaenoic acid (*ρ* = −0.94).

## Discussion

Tobacco leaf with interesting characteristics for animal nutrition, due to its high protein and active ingredients such as chlorogenic acid (Chen et al., 2007) and solanesol (Machado et al., 2010), which exhibited great value as feed resources for rabbits. Tobacco protein has been well documented in terms of amino acid profile and the efficiency ratio was higher in animal feed compared with other plant and animal protein (Fu et al., 2010). Kung et al. reported that tobacco leaf protein contained high levels of essential amino acids and could be used as excellent supplements for cereal diets consumed by world populations (Kung et al., 1980). Rao et al. also studied the antioxidant activity of tobacco leaf protein hydrolysates (Rao et al., 2007).

In this study, it was demonstrated that addition of LNT had no significant effect on the performance of meat rabbits. Similar to this study, Rossi et al. reported that the administration of tobacco seed cake did not impair the health status and growth performance of piglets (Rossi et al., 2013). However, the trend of ADG and FBW indicated that growth performance benefits from LNT addition during the whole experiment period. These results suggested that LNT can be developed as a feed resource without harm to the rabbit.

Slaughter performance including whole carcass weight and half carcass weight is one of the most important indicators of the economic benefits. During the growth process, deposition and distribution of fat in different parts caused differences in slaughter performance, which was closely related to the nutritional level of the diet. Serial studies reported the relationship between polyphenol and fat metabolism, and the dominant polyphenol in tobacco leaf were identified as chlorogenic acid and rutin (Silvester et al., 2019). Naveed reported chlorogenic acid inhibited fat accumulation in mice (Naveed et al., 2018). Wang et al. reported that rutin could reduce fat accumulation in *Caenorhabditis elegans* (Qin et al., 2021). Thus, in this study, the lower carcass weight may be related to the reduction of abdominal fat induced by the polyphenols in tobacco leaves.

Large amounts of articles had reported that phenolic and flavonoid compounds can decrease the total cholesterol and triglycerides levels. And, it was reported that tobacco leaves contain several active substances, such as chlorogenic acid and other flavonoid compounds (Zeng et al., 2022). Oral administration of chlorogenic acid could decrease the serum levels of TG and cholesterol (Hsu et al., 2021). In this experiment, the lower TG and cholesterol in the serum of meat rabbits may be related to the rich polyphenol content in tobacco. Liver is the main site of lipid metabolism and plays a very important role in the process of lipid absorption, synthesis, decomposition and transport. Changes in blood lipids often indicate changes in liver fat synthesis and decomposition (Everaert et al., 2022). The results obtained in this study indicated that LNT can regulate the lipid metabolism of rabbits and can be developed and utilized as a functional plant that promotes the health of animals.

Amount and proportion of blood composition changes could reflect the immune status of animals (Reece and Swenson, 2004). Lymphocyte and Eosinophil are important indicators of body’s immune function, and the increasing number represented enhancement of the immune resistance (Matsubara et al., 2005). Red blood cells are not only the medium for transporting oxygen and carbon dioxide, but also have certain immune functions. Based on the results in this study, we obtained that adding LNT to the rabbit diet could enhance rabbit immune status. Ding et al. (2019) reported that polysaccharides from the fruits of *Lycium barbarum* could regulate the immune response depending on the modulation of the gut microbiota in mice. Zhao et al. (2019) suggested that incorporating Mulberry (*Morus alba* L.) leaf polysaccharides into the diets of weaning pigs improves the immune functions of weaning pigs. Thus, the effects may due to the high polysaccharide in tobacco (Bai et al., 2018).

Rabbit meat contains high protein, high digestibility, low fat, low cholesterol and low calorie, which fulfilled with the needs of consumers (Dalle Zotte and Szendrő, 2011). Meat color, water preservation and other physical properties directly affected the quality and economic value of meat. And, obvious biochemical changes were occurred in meat after animal slaughtered. In this experiment, pH values were not significantly different, indicating that LNF addition had no effect on the pH value of rabbit meat. Meat color mainly depended on content of pigment myoglobin and hemoglobin. Both myoglobin and oxymyoglobin can be oxidized to methemoglobin by oxygen, which is in brown color (Lindahl et al., 2001). In this experiment, LNT addition increased the brightness of rabbit meat, indicating that adding LNT to the rabbit diet would increase antioxidant properties of rabbit meat.

Intestinal microbial community has become an important part of animals and serious studies have shown that changes in composition, ratio and diversity might be related to the occurrence of diseases. Rabbit contains high volume of cecum, which provided suitable conditions for microorganisms activities. Although there was no changes in microbial diversity in this study, LNT addition significantly changed the relative abundance of some taxa at the phylum, family and genus levels. Analysis of the relative abundance of bacterial communities revealed that the proportion of four genera *Eubacterium_siraeum_group*, *Monoglobus, Marvinbryantia* and *Alistipes,* and three families Rikenellaceae, Monoglobales and Ruminococcaceae were significantly enriched in LNT. Among the affected bacteria, *Eubacterium_siraeum_group* was proven to have the ability to ferment cellobiose to acetic acid (Zou et al., 2021). Monoglobus has a highly specialized pectin degrading glycobiome (Kim et al., 2019). *Eubacterium* could convert bile acids and cholesterol in the gut, promoting the body to keep homeostasis (Mukherjee et al., 2020). Ruminococcaceae is associated with promoting the regeneration of intestinal stem cells and preventing liver injury (Milton-Laskibar et al., 2022). Those results indicated LNT addition may be attributed to ferment carbohydrates and convert cholesterol to keep body healthy. Furthermore, we also found a negative correlation of *Eubacterium_siraeum_group* with the TG and CHO content in serum. Elevated gut microbiome abundance of *Rikenellaceae* is associated with reduced visceral adipose tissue and healthier metabolite profile (Tavella et al., 2021). Consisted with the above results, the lower cholesterol content and half carcass weight of rabbit supplemented with LNT may be related with these bacteria and lipid metabolism. Furthermore, genera *Marvinbryantia* was reported positively correlated with intestinal epithelial cell energy metabolism and butyrate production and previous studies have revealed that decrease of butyrate-producting in the gut microbiota is associated with the susceptibility of colorectal cancer (Wang et al., 2018). *Alistipes* is a relatively new genus of bacteria that is negatively correlated with the expression of inflammatory cytokines and could alleviate intestinal inflammation (Guo et al., 2021; Liu C. et al., 2022).We found that the abundance of *Alistipes* was positively correlated with the spleen and thymus weight which indicate increasing the immune status and negatively correlated with TG and CHO in serum. A negative association was found between several marks of liver damage with the *Ruminococcaceae* family (Milton-Laskibar et al., 2022). Hence, we propose that the increased abundance of *Marvinbryantia*, *Alistipes* and *Ruminococcaceae* indicated LNT addition significantly changes the bacterial microbiome of the cecum content to affect the intestinal function and body health. Further studies may focus on the study of multiple nonbacterial functions, leading to a deeper understanding of tobacco-host–microbe interactions.

The detected differential metabolites were screened and functionally annotated by the KEGG database. In the selected classifications, there were more predominantly accumulated lipid and amino acid metabolism and the enrichment metabolites mainly included lipid-like molecules, including fatty acid and conjugates and steroid conjugates. In agreement with previous studies, they have identified lipid and lipid-like molecules as metabolites of the fermented corn-soybean meal (Lu et al., 2019). Next, the bubble plot indicated that dietary addition of LNT significantly affected the metabolic pathways of phenylalanine metabolism and tyrosine metabolism. Phenylalanine and tyrosine metabolism have been associated with Alzheimer’s Disease related pathological changes, fat metabolism and immune response (Ueda et al., 2016; Rodman et al., 2019; Liu et al., 2021). These pathways have been associated with inflammation, metabolism, and immune response, suggesting that LNT addition acts through these pathways to have a benefit on the rabbit.

The results obtained in this study indicated that dietary addition of LNT significantly altered the metabolites in cecum digesta. We found that prostaglandin increased after LNT addition and it was positively correlated with *Eubacterium._siraeum_group* and thymus weight. Prostaglandin has been implicated in adipogenesis, being of white adipocytes and adipose tissue inflammation in obesity and insulin resistance (Deis et al., 2022). Hence, LNT addition may regulate lipid metabolism through increasing metabolites of Prostaglandin. Docosahexaenoic acid was regarded as a very important fatty acid for human health for many years, which was associated with alcohol syndrome, disorder and aggressive hostility and lipid disorders (Calder, 2016). In this study, Docosahexaenoic acid was increased in the LNT addition diet, and it was negatively associated with *Parabacteroides*. These results suggest that LNT addition may affect the metabolites including Prostaglandin and Docosahexaenoic acid which regulate lipid metabolism and protect health in rabbits. However, further studies are needed to clarify the specific mechanism of the interaction between gut microbita and metabolites.

## Conclusion

In summary, dietary LNT supplementation could decrease the serum concentration of triglycerides and cholesterol and increase the concentration of red blood cells and hemoglobin of rabbits, without affecting the rabbits’ growth performance. In addition, the results suggest that LNT addition altered the microbial composition and modulated the metabolic pathway of microbial metabolism in rabbit cecum. These alterations provide an alternative strategy for improving the health of rabbits. Overall, the results in this study indicate that LNT could be used as an effective feed resources.

## Data availability statement

The original contributions presented in the study are included in the article/Supplementary material, further inquiries can be directed to the corresponding authors.

## Ethics statement

The animal study was reviewed and approved by the Institutional Animal Care and Use Committee of Qingdao Agricultural University.

## Author contributions

CJ, JW, XY, YX, JZ, TT, JT, FJ, CW, YG, and YL performed the experiments, analyzed the results of the experiment, and prepared figures and tables. CJ, JW, ZZ, XY, and HZ conceived the study and wrote the manuscript. All authors have read and approved the final manuscript.

## Funding

This study was financially supported by the Agricultural Science and Technology Innovation Program (ASTIP-TRIC-ZD03, Elite youth program to HZ, ASTIP-TRIC05), Sichuan Tobacco Company (SCYC202014), and China Tobacco Company (110202103013).

## Conflict of interest

The authors declare that this study received funding from Sichuan Tobacco Company and China Tobacco Company. The funders were not involved in the study design, collection, analysis, interpretation of data, the writing of this article, or the decision to submit it for publication.

## Publisher’s note

All claims expressed in this article are solely those of the authors and do not necessarily represent those of their affiliated organizations, or those of the publisher, the editors and the reviewers. Any product that may be evaluated in this article, or claim that may be made by its manufacturer, is not guaranteed or endorsed by the publisher.

## Footnotes

^1^ https://www.mzcloud.org/

## Supplementary material

SUPPLEMENTARY FIGURE S1 Rarefaction curve of 16srna sequencing of cecal microorganisms. **(A)** Rarefaction curve of CK and LNT (TA) group cecal microbiological sequencing. **(B)** Rank Abundance of CK and LNT (TA) group cecal microbiological sequencing.

SUPPLEMENTARY FIGURE S2 Results of cecal microbiological diversity analysis in the CK and LNT (TA) groups. **(A)** Chao1 index box plot of microbial diversity in the CK group and LNT (TA) groups. **(B)** ACE index box plot of microbial diversity of the CK group and LNT (TA) groups. **(C)** Shannon index box plot of microbial diversity of the CK group and LNT (TA) groups. **(D)** Simpson index box plot of microbial diversity of the CK group and LNT (TA) groups. **(E)** The PCoA analysis of the CK and LNT (TA) groups. **(F)** The NMDS analysis of the CK and LNT (TA) groups.

SUPPLEMENTARY FIGURE S3 Heatmap of differential metabolites between CK and LNT (TT) groups, positive ion mode.

SUPPLEMENTARY FIGURE S4 Heatmap of differential metabolites between CK and LNT (TT) groups, neg ion mode.

## References

[ref1] Bai J. Jia X. Chen Y. Ning Z. Hu Z. Liu S.. (2018). Antioxidant activity and structural characterization of a new polysaccharide isolated from stem of flue-cured tobacco. J. Biol. Act. Prod. Nat. 8, 344–351. doi: 10.1080/22311866.2018.1541140

[ref2] Banožić M. Banjari I. Jakovljević M. Šubarić D. Tomas S. Babić J.. (2019). Optimization of ultrasound-assisted extraction of some bioactive compounds from tobacco waste. Molecules 24:1611. doi: 10.3390/molecules24081611, PMID: 31022850 PMC6514894

[ref3] Barreto G. E. Iarkov A. Moran V. E. (2015). Beneficial effects of nicotine, cotinine and its metabolites as potential agents for Parkinson’s disease. Front. Aging Neurosci. 6:340. doi: 10.3389/fnagi.2014.00340 25620929 PMC4288130

[ref5] Calder P. C. (2016). Docosahexaenoic acid. Ann. Nutr. Metab. 69, 8–21. doi: 10.1159/000448262 27842299

[ref6] Chen Y. Jimmy Yu Q. Li X. Luo Y. Liu H. (2007). Extraction and HPLC characterization of chlorogenic acid from tobacco residuals. Sep. Sci. Technol. 42, 3481–3492. doi: 10.1080/01496390701626677

[ref7] Dalle Zotte A. Szendrő Z. (2011). The role of rabbit meat as functional food. Meat Sci. 88, 319–331. doi: 10.1016/j.meatsci.2011.02.017, PMID: 21392894

[ref8] Deis J. Lin T. Y. Bushman T. Chen X. (2022). Lipocalin 2 deficiency alters prostaglandin biosynthesis and mTOR signaling regulation of thermogenesis and lipid metabolism in adipocytes. Cells 11:1535. doi: 10.3390/cells11091535, PMID: 35563840 PMC9105538

[ref9] Ding Y. Yan Y. Chen D. Ran L. Mi J. Lu L.. (2019). Modulating effects of polysaccharides from the fruits of Lycium barbarum on the immune response and gut microbiota in cyclophosphamide-treated mice. Food Funct. 10, 3671–3683. doi: 10.1039/C9FO00638A, PMID: 31168539

[ref120] Everaert N. Decuypere E. Buyse J. (2022). Adipose tissue and lipid metabolism. Stur. Av. Phys. 627–640.

[ref10] Fu H. Machado P. Hahm T. Kratochvil R. Wei C. Lo Y. (2010). Recovery of nicotine-free proteins from tobacco leaves using phosphate buffer system under controlled conditions. Bioresour. Technol. 101, 2034–2042. doi: 10.1016/j.biortech.2009.10.045, PMID: 19932614

[ref11] Guo C. Wang Y. Zhang S. Zhang X. Du Z. Li M.. (2021). Crataegus pinnatifida polysaccharide alleviates colitis via modulation of gut microbiota and SCFAs metabolism. Int. J. Biol. Macromol. 181, 357–368. doi: 10.1016/j.ijbiomac.2021.03.137, PMID: 33774071

[ref12] Hsu Y. W. Chen Y. Y. Tsai C. F. (2021). Protective effects of chlorogenic acid against carbon tetrachloride-induced hepatotoxicity in mice. PRO 10:31. doi: 10.3390/pr10010031

[ref13] Karlsson J. O. Röös E. (2019). Resource-efficient use of land and animals—environmental impacts of food systems based on organic cropping and avoided food-feed competition. Land Use Policy 85, 63–72. doi: 10.1016/j.landusepol.2019.03.035

[ref14] Kim C. C. Healey G. R. Kelly W. J. Patchett M. L. Jordens Z. Tannock G. W.. (2019). Genomic insights from Monoglobus pectinilyticus: a pectin-degrading specialist bacterium in the human colon. ISME J. 13, 1437–1456. doi: 10.1038/s41396-019-0363-6, PMID: 30728469 PMC6776006

[ref15] Kung S. Saunder J. A. Tso T. Vaughan D. A. Womack M. Staples R. C.. (1980). Tobacco as a potential food source and smoke material: nutritional evaluation of tobacco leaf protein. J. Food Sci. 45, 320–322. doi: 10.1111/j.1365-2621.1980.tb02605.x

[ref16] Lindahl G. Lundström K. Tornberg E. (2001). Contribution of pigment content, myoglobin forms and internal reflectance to the colour of pork loin and ham from pure breed pigs. Meat Sci. 59, 141–151. doi: 10.1016/S0309-1740(01)00064-X, PMID: 22062672

[ref17] Liu B. Cui Y. Ali Q. Zhu X. Li D. Ma S.. (2022). Gut microbiota modulate rabbit meat quality in response to dietary fiber. Front. Nutr. 9:849429. doi: 10.3389/fnut.2022.849429, PMID: 35392295 PMC8982513

[ref18] Liu C. Hua H. Guo Y. Qian H. Liu J. Cheng Y. (2022). Study on the hepatoprotective effect of Sporidiobolus pararoseus polysaccharides under the “gut microbiome-amino acids metabolism” network. Food Biosci. 49:101928. doi: 10.1016/j.fbio.2022.101928

[ref19] Liu P. Yang Q. Yu N. Cao Y. Wang X. Wang Z.. (2021). Phenylalanine metabolism is dysregulated in human hippocampus with alzheimer’s disease related pathological changes. J. Alzheimers Dis. 83, 609–622. doi: 10.3233/JAD-210461, PMID: 34334403

[ref20] Lu J. Zhang X. Liu Y. Cao H. Han Q. Xie B.. (2019). Effect of fermented corn-soybean meal on serum immunity, the expression of genes related to gut immunity, gut microbiota, and bacterial metabolites in grower-finisher pigs. Front. Microbiol. 10:2620. doi: 10.3389/fmicb.2019.02620, PMID: 31824447 PMC6879430

[ref22] Machado P. A. Fu H. Kratochvil R. J. Yuan Y. Hahm T. S. Sabliov C. M.. (2010). Recovery of solanesol from tobacco as a value-added byproduct for alternative applications. Bioresour. Technol. 101, 1091–1096. doi: 10.1016/j.biortech.2009.09.009, PMID: 19773155

[ref23] Matsubara T. Ichiyama T. Furukawa S. (2005). Immunological profile of peripheral blood lymphocytes and monocytes/macrophages in Kawasaki disease. Clin. Exp. Immunol. 141, 381–387. doi: 10.1111/j.1365-2249.2005.02821.x, PMID: 16045726 PMC1809464

[ref24] Milton-Laskibar I. Cuevas-Sierra A. Portillo M. P. Martínez J. A. (2022). Effects of resveratrol administration in liver injury prevention as induced by an obesogenic diet: role of ruminococcaceae. Biomedicine 10:1797. doi: 10.3390/biomedicines10081797, PMID: 35892696 PMC9330856

[ref25] Mukherjee A. Lordan C. Ross R. P. Cotter P. D. (2020). Gut microbes from the phylogenetically diverse genus Eubacterium and their various contributions to gut health. Gut Microbes 12:1802866. doi: 10.1080/19490976.2020.1802866, PMID: 32835590 PMC7524325

[ref26] Naveed M. Hejazi V. Abbas M. Kamboh A. A. Khan G. J. Shumzaid M.. (2018). Chlorogenic acid (CGA): a pharmacological review and call for further research. Biomed. Pharmacother. 97, 67–74. doi: 10.1016/j.biopha.2017.10.064, PMID: 29080460

[ref27] Qin X. Wang W. Chu W. (2021). Antioxidant and reducing lipid accumulation effects of rutin in Caenorhabditis elegans. Biofactors 47, 686–693. doi: 10.1002/biof.1755, PMID: 33988888

[ref28] Rao G. Zhao M. Lin W. Wang H. (2007). Antioxidative activity of tobacco leaf protein hydrolysates. Food Technol. Biotechnol. 45:80.

[ref29] Reece W. Swenson M. (2004). “The composition and functions of blood,” in Dukes’ Physiology of Domestic Animals. eds. Reece W. O. Erickson H. H. Goff J. P. Uemura E. E. (Ithaca FL: Cornell University Press), 26–52.

[ref30] Riljak V. Langmeier M. (2005). Nicotine an efficient tool of the neurobiological research today, the tool of treatment tomorrow. Prague Med. Rep. 106, 329–348. 16572927

[ref31] Rodman N. Martinez J. Fung S. Nakanouchi J. Myers A. L. Harris C. M.. (2019). Human pleural fluid elicits pyruvate and phenylalanine metabolism in Acinetobacter baumannii to enhance cytotoxicity and immune evasion. Front. Microbiol. 10:1581. doi: 10.3389/fmicb.2019.01581, PMID: 31379769 PMC6650585

[ref32] Rossi L. Dell’Orto V. Vagni S. Sala V. Reggi S. Baldi A. (2014). Protective effect of oral administration of transgenic tobacco seeds against verocytotoxic *Escherichia coli* strain in piglets. Vet. Res. Commun. 38, 39–49. doi: 10.1007/s11259-013-9583-9, PMID: 24249478

[ref33] Rossi L. Fusi E. Baldi G. Fogher C. Cheli F. Baldi A.. (2013). Tobacco seeds by-product as protein source for piglets. Open J. Vet. Med. 3, 73–78. doi: 10.4236/ojvm.2013.31012

[ref34] Ru Q. M. Wang L. J. Li W. M. Wang J. L. Ding Y. T. (2012). In vitro antioxidant properties of flavonoids and polysaccharides extract from tobacco (*Nicotiana tabacum* L.) leaves. Molecules 17, 11281–11291. doi: 10.3390/molecules170911281, PMID: 23001388 PMC6268702

[ref35] Sifola M. I. Carrino L. Cozzolino E. del Piano L. Graziani G. Ritieni A. (2021). Potential of pre-harvest wastes of tobacco (*Nicotiana tabacum* L.) crops, grown for smoke products, as source of bioactive compounds (phenols and flavonoids). Sustainability 13:2087. doi: 10.3390/su13042087

[ref36] Silvester A. J. Aseer K. R. Yun J. W. (2019). Dietary polyphenols and their roles in fat browning. J. Nutr. Biochem. 64, 1–12. doi: 10.1016/j.jnutbio.2018.09.028, PMID: 30414469

[ref37] Tabe L. Higgins T. (1998). Engineering plant protein composition for improved nutrition. Trends Plant Sci. 3, 282–286. doi: 10.1016/S1360-1385(98)01267-9

[ref38] Tavella T. Rampelli S. Guidarelli G. Bazzocchi A. Gasperini C. Pujos-Guillot E.. (2021). Elevated gut microbiome abundance of *Christensenellaceae, Porphyromonadaceae* and *Rikenellaceae* is associated with reduced visceral adipose tissue and healthier metabolic profile in Italian elderly. Gut Microbes 13, 1–19. doi: 10.1080/19490976.2021.1880221, PMID: 33557667 PMC7889099

[ref39] Ueda K. Nakamura Y. Yamaguchi M. Mori T. Uchida M. Fujita S. (2016). Amino acid mixture enriched with arginine, alanine, and phenylalanine stimulates fat metabolism during exercise. Int. J. Sport Nutr. Exerc. Metab. 26, 46–54. doi: 10.1123/ijsnem.2015-0137, PMID: 26252574

[ref400] Wang J. Bian S. Zhang J. Ju F. Tian T. Xiao Y.. (2022). Low-nicotine tobacco pedigree breeding and leaf component Analyses. Mol. Plant. Bre. 6, 1945–1954.

[ref40] Wang J. Qin C. He T. Qiu K. Sun W. Zhang X.. (2018). Alfalfa-containing diets alter luminal microbiota structure and short chain fatty acid sensing in the caecal mucosa of pigs. J. Anim. Sci. Biotechnol. 9, 1–9. doi: 10.1186/s40104-017-0216-y 29372054 PMC5769528

[ref41] Xu K. Du X. Ren X. Li X. Li H. Fu X.. (2022). Structural modifications and biological activities of natural α-and β-Cembrenediol: a comprehensive review. Pharmaceuticals 15:601. doi: 10.3390/ph15050601, PMID: 35631427 PMC9143853

[ref42] Zeng G. Ran Y. Huang X. Li Y. Zhang M. Ding H.. (2022). Optimization of ultrasonic-assisted extraction of chlorogenic acid from tobacco waste. Int. J. Environ. Res. Public Health 19:1555. doi: 10.3390/ijerph19031555, PMID: 35162594 PMC8835221

[ref43] Zhao X. Yang R. Bi Y. Bilal M. Kuang Z. Iqbal H. M.. (2019). Effects of dietary supplementation with mulberry (*Morus alba* L.) leaf polysaccharides on immune parameters of weanling pigs. Animals 10:35. doi: 10.3390/ani10010035, PMID: 31878017 PMC7022547

[ref44] Zou Y. Liang N. Zhang X. Han C. Nan X. (2021). Functional differentiation related to decomposing complex carbohydrates of intestinal microbes between two wild zokor species based on 16SrRNA sequences. BMC Vet. Res. 17, 1–12. doi: 10.1186/s12917-021-02911-z 34116670 PMC8196462

