# Supplementary figures and images for: Investigation of the growth performance, blood status, gut microbiome and metabolites of rabbit fed with low-nicotine tobacco
Source: Front Microbiol. 2022 Oct 13;13:1026680. doi: 10.3389/fmicb.2022.1026680 (PMC9615924; doi:10.3389/fmicb.2022.1026680)

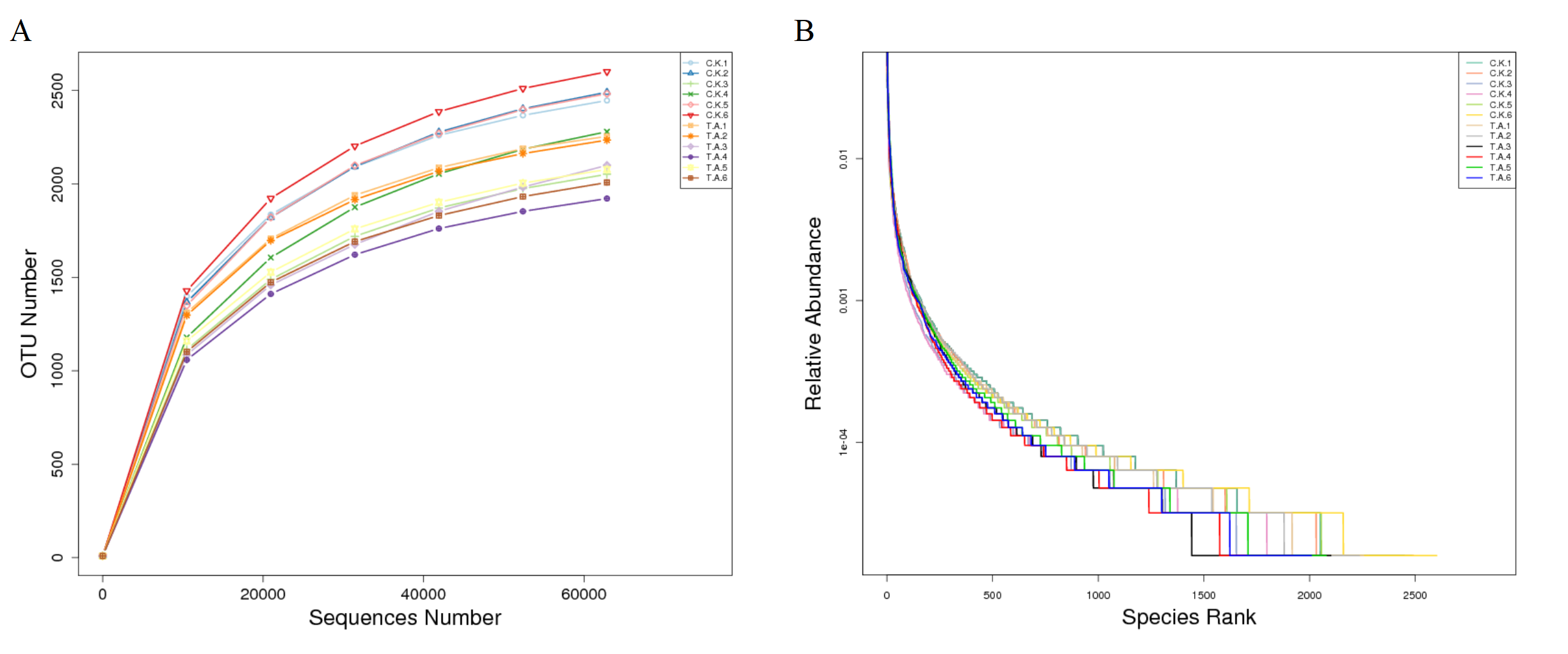

Supplement: SUPPLEMENTARY FIGURE S1 — Rarefaction curve of 16srna sequencing of cecal microorganisms. (A) Rarefaction curve of CK and LNT (TA) group cecal microbiological sequencing. (B) Rank Abundance of CK and LNT (TA) group cecal microbiological sequencing. [file Image_1.TIF]

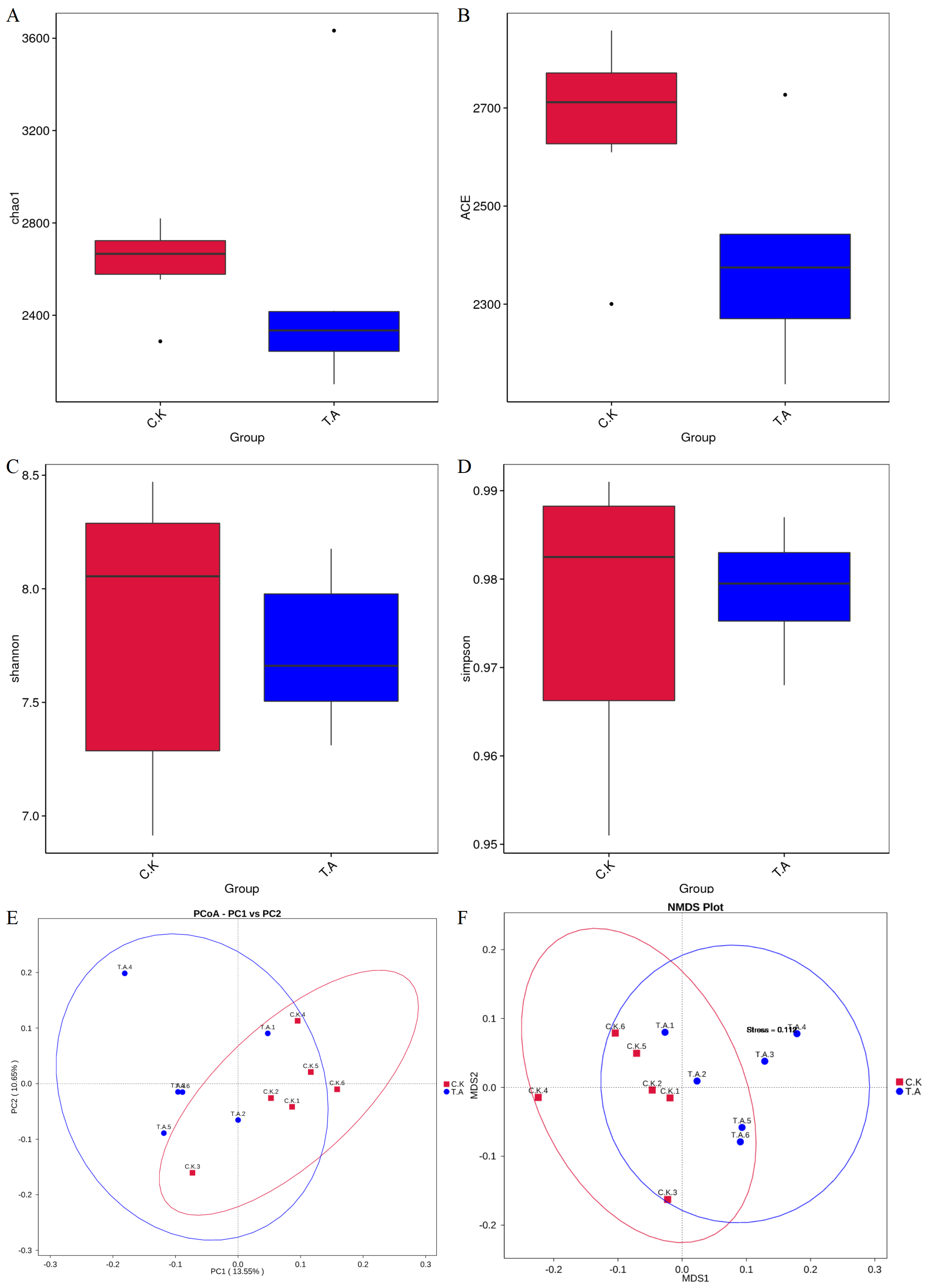

Supplement: SUPPLEMENTARY FIGURE S2 — Results of cecal microbiological diversity analysis in the CK and LNT (TA) groups. (A) Chao1 index box plot of microbial diversity in the CK group and LNT (TA) groups. (B) ACE index box plot of microbial diversity of the CK group and LNT (TA) groups. (C) Shannon index box plot of microbial diversity of the CK group and LNT (TA) groups. (D) Simpson index box plot of microbial diversity of the CK group and LNT (TA) groups. (E) The PCoA analysis of the CK and LNT (TA) groups. (F) The NMDS analysis of the CK and LNT (TA) groups. [file Image_2.TIF]

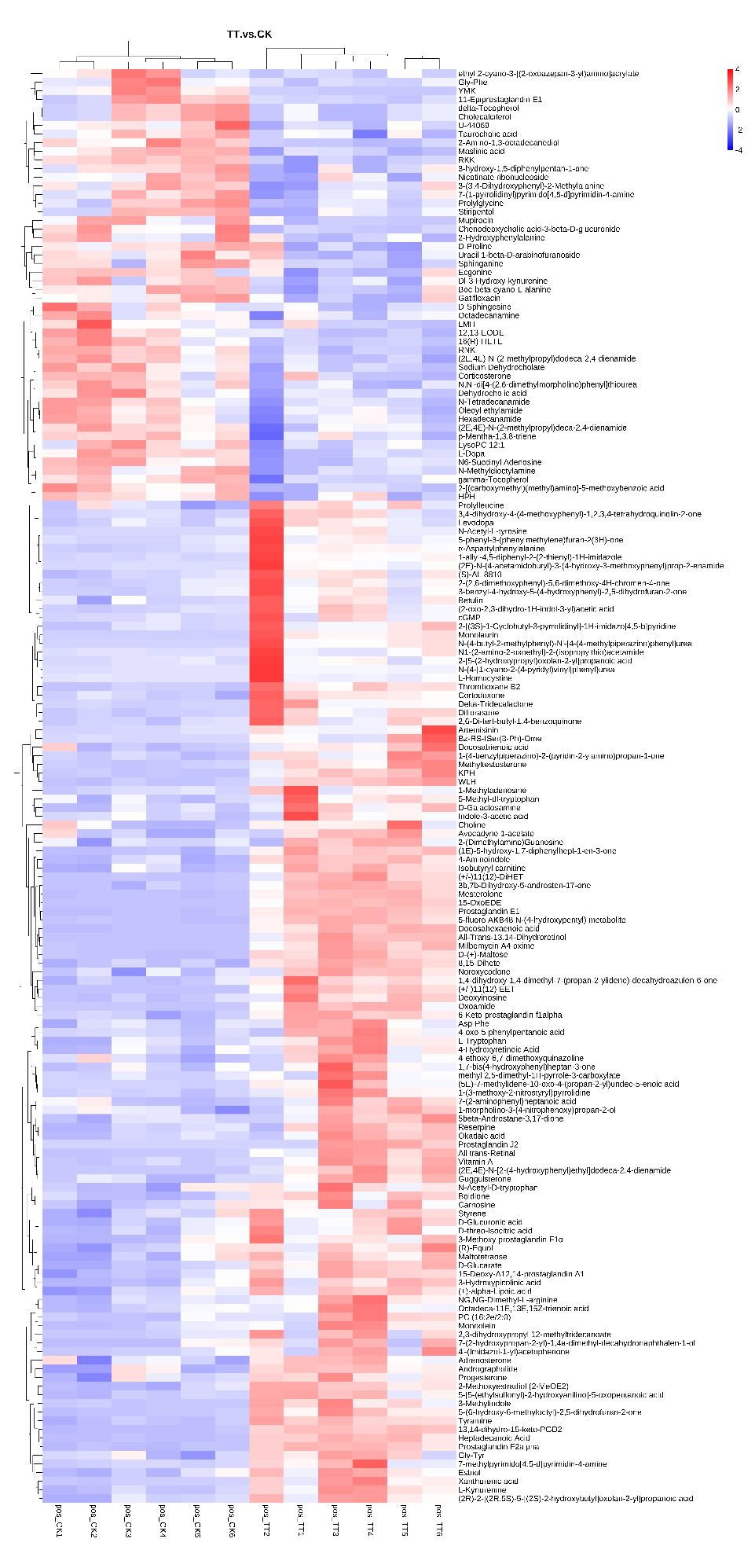

Supplement: SUPPLEMENTARY FIGURE S3 — Heatmap of differential metabolites between CK and LNT (TT) groups, positive ion mode. [file Image_3.TIF]

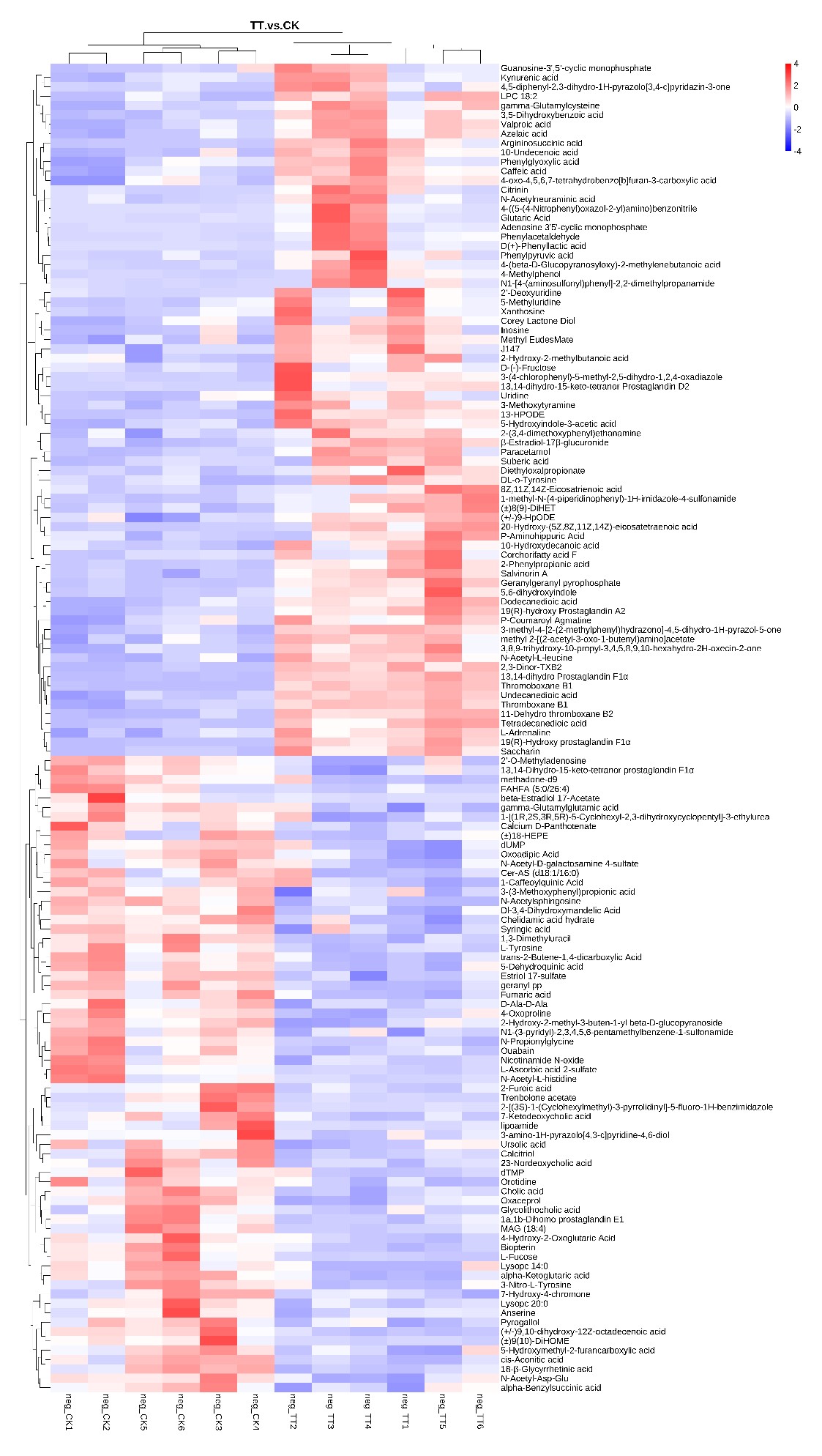

Supplement: SUPPLEMENTARY FIGURE S4 — Heatmap of differential metabolites between CK and LNT (TT) groups, neg ion mode. [file Image_4.JPEG]
