# Supplementary material for: Investigation of the growth performance, blood status, gut microbiome and metabolites of rabbit fed with low-nicotine tobacco
Source: Front Microbiol. 2022 Oct 13;13:1026680. doi: 10.3389/fmicb.2022.1026680 (PMC9615924; doi:10.3389/fmicb.2022.1026680)
Supplement: Supplementary file 5 [file Data_Sheet_1.docx]

**Supplementary TABLE**

**TABLE S1.**

Determination of components in the leaves of low-nicotine tobacco

| Determination of components | Content /% |
| --- | --- |
| Protein /% | 14.35 |
| Amino acid /% | 1.85 |
| Total sugar /% | 21.95 |
| Coarse fiber /% | 8.13 |

**TABLE S2.**

Composition and nutrient levels of experimental diets (air-dry basis)

| Recipe | Tobacco leaf addition level /% | | | |
| --- | --- | --- | --- | --- |
|  | 0 | 5 | 10 | 20 |
| Material | | | | |
| Germ meal | 13.00 | 8.00 | 9.00 | 12.00 |
| Corn | 13.00 | 16.00 | 15.00 | 15.00 |
| Peanut seedlings | 30.00 | 25.00 | 20.00 | 8.00 |
| Bean pulp | 15.00 | 16.00 | 15.00 | 13.00 |
| Rice husk | 6.00 | 8.00 | 8.00 | 10.00 |
| Bran | 18.00 | 17.00 | 18.00 | 17.00 |
| LNT | 0.00 | 5.00 | 10.00 | 20.00 |
| Mixture | 5.00 | 5.00 | 5.00 | 5.00 |
| Total | 100.00 | 100.00 | 100.00 | 100.00 |
| Nutrition | | | | |
| De /(MJ/kg) | 13.15 | 13.28 | 13.36 | 13.24 |
| Cf | 12.99 | 12.87 | 13.01 | 12.82 |
| Ndf | 36.74 | 35.25 | 36.22 | 37.78 |
| Adf | 19.85 | 18.96 | 19.25 | 19.55 |
| Cp | 16.78 | 16.71 | 16.69 | 16.73 |
| Tp | 0.49 | 0.48 | 0.52 | 0.47 |
| Ca | 0.93 | 0.87 | 0.95 | 0.91 |

The premix provided the following per kg the diet: Fe 100 mg, Cu 20 mg, Zn 90 mg, Mn 30 mg, Mg 150 mg, VA 4000 IU, VD3 1000 IU, VE 50 mg, choline chloride 1 g.
